# Core Values in the Traditional Provision of Hearing Health Care

**DOI:** 10.1044/2022_JSLHR-22-00540

**Published:** 2023-02-07

**Authors:** Katherine N. Menon, Michelle Hoon-Starr, Katie Shilton, Eric C. Hoover

**Affiliations:** a Department of Hearing and Speech Sciences, University of Maryland, College Park; b College of Information Studies, University of Maryland, College Park

## Abstract

**Purpose:**

Untreated hearing loss is a significant public health issue affecting the quality of life of millions of Americans. Barriers to treatment invite novel and innovation solutions, but as these solutions create new treatment delivery models, they also may—purposefully or accidentally—challenge the values of the field.

**Method:**

Value-sensitive design methodology is used in this study to identify the values in current hearing health care service delivery. We performed qualitative content analysis of questionnaires, clinical practice guidelines, and professional ethics documents that represent the intended and enacted values in audiology.

**Results:**

The result is a comprehensive list of values that can be used as a structured codebook for systematic textual analysis of materials representing current best practices in the provision of hearing health care services. A secondary result is an analysis of the relative importance of values in audiology, inferred from the frequency of references to each value.

**Conclusions:**

Subjective benefit, professional duties, and self-efficacy were the core values identified in the current provision of audiologic care, and these values should be central to considerations for new hearing health care models and technologies.

Hearing loss is a serious public health problem that affects an estimated two thirds of adults over the age of 70 years (Bainbridge & Wallhagen, 2014). In the coming years, more people will be affected by hearing loss as the population ages; an estimated 41 million older Americans will suffer from hearing loss by 2060 (Nieman & Lin, 2017). Untreated hearing loss has been reported to negatively impact quality of life (Ciorba et al., 2012) and has been associated with decreased independence (Bainbridge & Wallhagen, 2014), increased social isolation (Vas et al., 2017), increased risk of falls (Lin & Ferrucci, 2012), accelerated cognitive decline (Nieman & Lin, 2017), and other adverse health conditions. The widespread implementation of preventative measures against COVID-19, including masking and social distancing, has led to additional communication barriers for those who are hard of hearing (Trecca et al., 2020). Untreated hearing loss also presents a substantial economic burden to both the individual and society. It has been estimated that insured individuals who are hard of hearing in the United States are burdened with 20% higher total health care–related payments over an 18-month period (Simpson et al., 2018), and total health care costs tend to be 46% higher over a 10-year period when comparing adults with untreated hearing loss to those without hearing loss (Reed et al., 2019). Moreover, modeling indicates that reducing the prevalence of hearing loss by 20% would produce an economic benefit ranging between $58 billion and $152 billion annually (Neitzel et al., 2017).

Despite the high prevalence of hearing loss and its associated negative effects, significant disparities exist in treatment. Few people who could benefit from treatment seek it; less than 30% of adults over 70 years old who would benefit from a hearing aid report using one (Dammeyer et al., 2017), and the average delay from diagnosis to treatment is 8.9 years (Simpson et al., 2019). Socioeconomic, racial/cultural, and geographical barriers mean that these disparities disproportionately impact marginalized groups (Barnett et al., 2017; Ingram et al., 2016; Nieman et al., 2016; Powell et al., 2019). In rural communities, access to hearing health care is limited by the travel distance to the nearest provider (Bush, 2021; Coco et al., 2018). Both patients and health care providers living in areas with low health care access tend to have poorer awareness of the benefits associated with the diagnosis and treatment of hearing loss (Bush et al., 2015).

There is a long history of research exploring reasons behind the apparent preference for nonuse of hearing aids among candidates for amplification. Commonly cited differences between cohorts of hearing aid users and nonusers include degree of hearing loss, personal feelings about hearing aid use, ability to pay the cost of devices and associated treatment, comfort with wearing and using devices, expectations about outcomes, and social support (Garstecki & Erler, 1998). More recent work has focused on characterizing the psychosocial and cognitive factors that impact hearing aid uptake. Adults with better psychomotor function and poorer self-reported health status are more likely to adopt and use their hearing aids (Nixon et al., 2021). The subjective experience of hearing also influences help-seeking behavior; Humes and Dubno (2021) found that older adults who do not seek treatment for hearing loss tend to perceive less difficulty with communication and are generally less accepting of their condition compared with their treatment-seeking peers. Recent high-profile critiques exploring underutilization of hearing health care services cite access to a hearing health care professional and cost of devices as the largest barriers to treatment (National Academies of Sciences, Engineering, and Medicine [NASEM], 2016; President's Council of Advisors on Science and Technology [PCAST], 2015).

The PCAST and NASEM reports proposed an over-the-counter (OTC) hearing aid model as a solution. In 2017, legislation was passed that introduced a new class of amplification devices to be sold directly from manufacturer to consumer (Over-the-Counter Hearing Aid Act of 2017, passed as part of the Food and Drug Administration Reauthorization Act of 2017, HR 2430, § 934). While OTC is likely to address issues of cost and access related to devices, its implementation could also represent a shift away from the professional rehabilitative services associated with audiology and successful hearing aid outcomes. Remote service delivery (teleaudiology) is another solution that has been widely implemented to improve access to care. Teleaudiology can increase access to crucial services for vulnerable populations that experience barriers to attending clinic appointments (Muñoz et al., 2021). Teleaudiology can reduce the costs associated with traveling long distances for multiple appointments and of building and staffing comprehensive audiology clinics in remote areas. However, this solution may not address other barriers associated with treatment for hearing loss, such as device costs and stigma.

There is a need to compare different solutions in terms of their capability to help underserved individuals overcome barriers to treatment. With a better understanding of the differences in competing models, we might mitigate unintended consequences that could negatively affect patients and consumers. This study analyzed traditional, clinical audiology to identify what is considered important in the current dominant model of hearing health care service delivery. Specifically, this study aimed to evaluate hearing health care as it pertains to the epidemic of untreated hearing loss. According to the Office of Disease Prevention and Health Promotion (n.d.), an objective of the Healthy People 2030 initiative is to “increase the proportion of adults with hearing loss who use a hearing aid.” In this study, qualitative research methods were used to evaluate the values of traditional audiology in order to develop a tool that will allow us to compare differences between service delivery models that provide hearing aids for adult patients who could benefit from amplification.

Value-sensitive design (VSD) is an approach used to describe, consider, evaluate, or proactively design for social values supported by a technology, product, or service (Friedman et al., 2002, 2013). For example, values analyses have been used in social science research on technology to develop solutions that identify gaps between intended and enacted values and preserve the values important to stakeholders involved (Shilton, 2018; Shilton et al., 2014). Such analyses often utilize qualitative content analysis techniques, which can help researchers interpret meaning from the content of textual data (Hsieh & Shannon, 2005; Knudsen et al., 2012). Although VSD has been used to design, develop, and implement health information systems (Walton & DeRenzi, 2009) and characterize values of stakeholders with multiple chronic health conditions (Berry et al., 2018), VSD has not been applied to hearing health care or the provision of prosthetic technologies like hearing aids and cochlear implants.

In VSD, the first step is to identify *what* values are and *where* they can be found (Shilton et al., 2014). Schwartz (1994) lists five features of the definition of values for which there is widespread agreement in the literature: “A value is a (1) belief (2) pertaining to desirable end states or modes of conduct, that (3) transcends specific situations, (4) guides selection or evaluation of behavior, people, and events, and (5) is ordered by importance relative to other values to form a system of value priorities.” Though values can be defined in various ways, a helpful starting definition is *principles or qualities appreciated as important by an individual or system*. Questions of where values can be observed can be guided by thinking about spectra along which values might influence either technology design or service delivery (Shilton et al., 2014). Shilton et al. (2014) suggest that *intention* and *enactment* are two separate dimensions of values that should be considered in design research. Values can differ in intention, as they can vary from purposive to accidental. An audiologist may incorporate real-ear measurements in their routine clinical practice, representing values of accuracy and evidence-based practice. An audiologist might fit patients with Bluetooth-enabled devices and unintentionally facilitate Bluetooth-based location tracking that sacrifices the value of privacy. Values can also differ in enactment, either performed or potential. An audiologist might walk a hearing aid user through volume adjustment, performing values of comfort or customization. In contrast, features that are not explained by the audiologist and never used, such as the ability to connect a smartphone to change programs, represent a potential for the value of control that was never enacted.

The values appreciated as important by hearing health care can be found in numerous places. We focused on values dimensions of intention and enactment to guide our selection of source material for this study. We aimed to collect and analyze textual documents where the spectra of both intended and enacted values in clinical audiology could be identified, focusing primarily on documents that describe the provision of hearing health care services for adults who could benefit from hearing aids or rehabilitative audiological services. Three categories of documents were selected: questionnaires, clinical practice guidelines (CPGs), and codes of ethics. Questionnaires represent *enacted* values in that audiologists use them to assess outcomes and determine treatment success. CPGs are a record of what audiologists do, following best-practice recommendations where available, and, therefore, document both dimensions of intended and enacted values in clinical practice. Codes of ethics represent *intended* values that define moral behavior for audiologists, as determined by professional organizations. Our goal was to characterize the values found in this corpus to compose a comprehensive list of the intended and enacted values in hearing health care service delivery. A secondary goal was to analyze the frequency that each value was expressed as a proxy for the relative importance of different values. Identifying these values will facilitate comparisons between competing models and guide the development of novel approaches that preserve stakeholder values.

## Method

A three-step qualitative content analysis approach was used to create a comprehensive list of the values in hearing health care. Our research goals guided our choice of qualitative research design and techniques (Knafl & Howard, 1984). We adapted the directed content analysis methods described by Hsieh and Shannon (2005) to achieve our goal of characterizing and extending the conceptual framework of values in hearing health care.

*Step 1: Identify source material representing the intended and enacted values in hearing health care.* We collected textual documents that describe the provision of audiological services for American adult patients who could benefit from treatment in the form of amplification devices or aural rehabilitation services. A literature review was conducted to collect potential questionnaires representing enacted values in the provision of audiological services. The electronic database PubMed/MEDLINE and the search engine Google Scholar were used for searches of questionnaires addressing adults with hearing difficulty or hearing aids. Databases were searched for peer-reviewed articles containing outcome questionnaire batteries for hearing health care services published between 1960 and 2020 using the search term “outcome assessment” in conjunction with each of the following terms: “hearing aid,” “audiology,” “hearing,” “aural rehabilitation,” and “communication.” The search was conducted in August 2020 and updated in February 2021. Questionnaires were included in the study if they were published between January 1960 and January 2020, were written in English, and contained a complete corpus of outcome questions. Questionnaires were excluded if they were published prior to January 1960, were written in a language other than English, or did not contain outcome-related questions. In total, the search generated 43 hits. After questionnaires that did not meet the inclusion criteria were excluded, a total of 24 questionnaires were chosen for analysis. Another literature review was conducted to collect CPGs. CPGs exist for various services; however, we targeted those addressing treatment of adult patients with hearing aids. To collect relevant CPGs, we conducted “hand searches” of professional organization websites relevant to audiology. Key professional organizations included the American Academy of Audiology (AAA), the American Speech-Language-Hearing Association (ASHA), The Independent Hearing Aid Fitting Forum, and the Audiology Practice Standards Organization. One code of ethics was obtained from each of the websites of these four professional organizations, with the exception of AAA where two CPGs were elicited. To collect relevant codes of ethics, we conducted another hand search of professional organization websites relevant to audiology. Key professional organizations included AAA, the Academy of Doctors of Audiology, ASHA, and the National Board for Certification in Hearing Instrument Sciences. One code of ethics document was obtained from each of the websites of these four professional organizations. Our final corpus included a total of 33 relevant documents to be analyzed for values. We collected five CPGs, four codes of ethics, and 24 questionnaires (listed in the Appendix).

*Step 2: Create the preliminary codebook.* In our directed content analysis approach, theory and relevant search findings served as guidance to compile the initial list of values (Hsieh & Shannon, 2005; Mayring, 2004). In this structured, top-down phase, group members collaboratively brainstormed possible values based on expertise in values research and audiology. To establish a framework for sorting values into meaningful groups, we determined three different categories of potential values (Atkinson & Coffey, 1995; Patton, 2002). These broad categories consisted of instrumental, patient use, and moral values. Instrumental values capture logistical or operational concerns, patient use values capture the attributes that shape the patient experience, and moral values capture ethical concerns. We listed key concepts in each category to serve as our initial codebook of values and agreed upon operational definitions for each code before data analysis (Potter & Levine-Donnerstein, 1999).

*Step 3: Refine the codebook.* The next step in our directed content analysis consisted of a bottom-up phase that involved utilizing the preliminary codebook to analyze the collected documents from Step 1. The critical part of this phase was searching for concepts that could not be captured with our initial list of values. In this step, values were derived directly from source material through an iterative coding process by symbolically assigning meaning to text data (Locke et al., 2020; Saldaña, 2009). Coders read documents to identify values listed in the initial codebook and to search for values that did not fit into our existing coding scheme (Miles & Huberman, 1994; Morgan, 1993; Morse & Field, 1995). Coding was performed in NVivo 12 Pro (QSR International Pty. Ltd., 2020), a computer software package for the comprehensive analysis of unstructured documents. NVivo facilitates the coding of textual documents by allowing users to “tag” and subsequently categorize segments of text to analyze coded data to identify trends and patterns. The preliminary codebook was revised through this process by adding new values identified in the source material and by revising the delineation of values. Initially, documents were analyzed by two coders together until they subjectively agreed about the interpretation of values. Subsequently, documents were coded by one coder except when input was requested from a second coder to resolve the coding of text that was ambiguous or difficult to interpret. Throughout the coding process, examples of each value were added to the codebook to ease and standardize the coding process, listed in Table 1.

**Table 1.** Codebook of values in hearing health care.

Value	Definition	Examples of how value might appear	Pull quote from source material
**Instrumental values: logistical or operational concerns**			
Efficiency	Use of time	Appointment number, duration; time required to self-fit or adjust fitting	“The hearing aid orientation process begins with the initial hearing aid fitting and may continue over several visits. Hearing aid orientation is complete only when all appropriate information has been provided and the patient (or family member/caregiver) is competent to handle the instruments or declines further postfitting care.” Guideline for audiologic management of the adult patient (Valente, 2006)
Cost	Financial considerations	Related to money, warranty, price of devices, payer	“Members must provide full disclosure of the fees/prices of products and services. This information must be disclosed by providing a comprehensive schedule of fees to the patient, to the best extent possible, in advance of providing services and products.” Code of ethics (Academy of Doctors of Audiology, n.d.)
Accuracy	Measured quantity in good agreement with reality	Fit-to-target, sensitivity and specificity, electroacoustic tolerances, calibration, ambient noise, noise floor, frequency response, comparison to normative data, standards compliant	“An assessment of initial product quality is completed, using standard electroacoustic measures to verify either manufacturer or published specifications.” Hearing aid fitting standards for adult and geriatric patients (Audiology Practice Standards Organization, 2021)
Safety	Prevention of physical harm	Choking hazard, environmental awareness (omni mic, fitting minimal benefit), output limits, hygiene/infection control	“Question 20. Can you hear warning signals, such as automobile horns, railway crossing bells, or emergency vehicle sirens?” Scale for Self-Assessment of Hearing Handicap
Objective benefit	Benefit from treatment measured as improved performance on a task	Measured improvement in audibility, speech intelligibility, speech in noise; directional benefit	“Objective outcomes often refer to measures of improved speech understanding in various everyday listening situations.” Guideline for audiologic management of the adult patient (Valente, 2006)
Evidence-based	Supported by scientific research	Procedures and devices evaluated in peer-reviewed publications, evidence-based practice and treatment	“Audiologists apply research findings and quality improvement measures to develop or revise local clinical policies, procedures and clinical pathways to improve patient care.” Standards of practice for audiology (American Academy of Audiology, 2019)
Design	Physical or aesthetic characteristics	Form-factor, aesthetics, packaging	“Technological advances in hearing aid design and selection and maturation of the audiology profession have led to significant improvements in the fitting of hearing aids over the past 2 decades.” Guidelines for hearing aid fitting for adults (ASHA Ad Hoc Committee on Hearing Aid Selection and Fitting, 1998)
**Patient use values: attributes that shape the patient experience**			
Health	Diagnosis and/or referral for medical conditions	Medical referral, exposure to dangerous intensities, accurate characterization of impairment, ototoxic monitoring, cerumen management	“As a result of the audiologic assessment, the individual may be referred for additional services (e.g., electrophysiologic tests, medical or surgical intervention) before further audiologic management.” Guidelines for hearing aid fitting for adults (ASHA Ad Hoc Committee on Hearing Aid Selection and Fitting, 1998)
Ease of use	Facilitating device–user interaction	Related to effort, training the user, reading documentation, following instructions, required abilities (e.g., insertion, maintenance), behavioral reinforcement, frequency of repairs, troubleshooting	“Orientation is device- and patient-centered and includes use, care, and maintenance of the hearing aid(s) and accessories.” Hearing aid fitting standards for adult and geriatric patients (Audiology Practice Standards Organization, 2021)
Comfort	Absence of pain or constraint	Physical fit, loudness, comfort in noise, occlusion, feedback	“Hearing aid fitting and verification procedures are expected to yield a comfortable fit of hearing aids including all desired features.” Guideline for audiologic management of the adult patient (Valente, 2006)
Subjective benefit	Benefit from treatment that is perceived by patient	Improvement in audibility, intelligibility, localization, clarity, sound quality; reduction in activity limitation; decrease of participation restriction; perception of self; reduction of listening effort	“4. I have difficulty hearing a conversation when I'm with one of my family at home.” “5. I have trouble understanding the dialogue in a movie or at the theater.” The Abbreviated Profile of Hearing Aid Benefit (Cox & Alexander, 1995)
Self-efficacy	Ability to act as one's own advocate	Disclosure of hearing loss, visibility of hearing loss, use of communication strategies; self-advocacy; confidence	“76. You are at a restaurant. When you miss something important that the waitress/ waiter said, do you ask for it to be repeated? 77. You are talking with a close friend. When you miss something important that was said, do you immediately adjust your hearing aid to help you hear better?” Hearing Performance Inventory (Giolas et al., 1979)
Satisfaction	Meeting user needs and desires	Customer service; decision to keep devices	“Question 4. Considering everything, do you think your hearing aids are worth the trouble?” The International Outcome Inventory for Hearing Aids (IOI-HA) : Psychometric Properties of the English Version (Cox & Alexander, 2002)
**Moral values: principles defining good behavior**			
Access to care	Patient's ability to obtain diagnostics, devices, treatment, and services	Availability of providers, ability to purchase devices, appropriate referrals, discontinuance of care, telepractice	“Audiologists are responsible for developing, implementing and monitoring the success of follow-up protocols to ensure that individuals identified through screening efforts are referred for further assessment and treatment.” Standards of practice for audiology (American Academy of Audiology, 2019)
Privacy	Protection of personal information	Appropriate collection and use of health information, secure storage, confidentiality, data minimization, notice and consent, data retention, data logging by devices, medical records	“Members shall not release professional and personal information obtained from the patient without the written permission of the patient in accordance with applicable state and federal law.” Code of ethics (Academy of Doctors of Audiology, n.d.)
Autonomy	Patient's ability to act independently	Independence, healthy aging, promote self-efficacy, informed consent, patient education	“Individuals shall provide persons served with the information a reasonable person would want to know about the nature and possible effects of services rendered or products provided or research being conducted.” Standards of practice for audiology (American Academy of Audiology, 2019)
Equity	Fairness in treatment or outcomes	Nondiscrimination, accommodation, cultural and linguistic diversity, stigma	“Individuals shall not discriminate in the delivery of professional services or in the conduct of research and scholarly activities on the basis of race, ethnicity, sex, gender identity/gender expression, sexual orientation, age, religion, national origin, disability, culture, language, or dialect.” Code of ethics (American Speech-Language-Hearing Association, 2016)
Professional duties	Licensed provider's adherence to standards for professional conduct	Scope of practice; conflict of interest (financial, professional, research), truthfulness, competence, continuing education, lawfulness, mentoring/supervision, managing differences of professional opinion, maintenance of records/documentation, collaboration with other professionals	“Member's clinical judgment and practice must not be determined by economic interest in, commitment to or benefit from, professionally related commercial enterprises.” Code of ethics (Academy of Doctors of Audiology, n.d.)

## Results

The primary goal of this study was to identify the values of hearing health care service delivery as expressed by documents integral to the practice of audiology. The result is a structured codebook of values identified in hearing health care shown in Table 1. We created an iterative taxonomy of values that roughly divided the list into three categories: instrumental, patient use, and moral. Instrumental values included accuracy, cost, design, efficiency, evidence-based, objective benefit, and safety. Instrumental values were primarily found in CPGs, which focused on accurate, objective assessment of hearing ability and intervention success. Patient use values included comfort, ease of use, health, satisfaction, self-efficacy, and subjective benefit. Patient use values were primarily found in questionnaires, which commonly included questions about the patient's perceived abilities and limitations before and after intervention. Moral values included access to care, autonomy, privacy, equity, and professional duties. Moral values were primarily found in codes of ethics documents, consistent with the goal of those documents to dictate the ethical guidelines of the practice of audiology.

**Figure 1.**
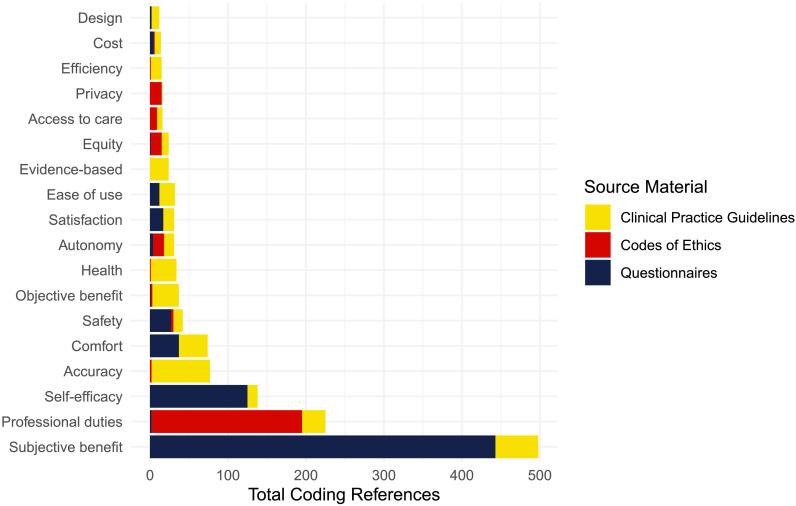
Incidence of each coding reference representing values in hearing health care service delivery.

A secondary goal of the study was to estimate the relative importance of different values overall and within each category of documents. We used rank order comparisons of the frequency of each value coded in the selected documents to operationalize relative importance (Curtis et al., 2001; Hsieh & Shannon, 2005). Figure 1 shows the number of coding references for each value identified in the collected documents. Sorted values demonstrated an exponential decrease in total coding references. Subjective benefit was the value most often identified in the source material overall, with 493 coding references. The instrumental value coded most often was accuracy, and the patient use value coded most often was subjective benefit. The moral value coded most often was professional duties, but the number of coding references was inflated by the fact that this value encapsulated the long list of specific codes of conduct expressed by the professional societies' codes of ethics documents. The next moral value coded most often was autonomy, a value that was well represented in all three types of documents coded. Table 2 shows rankings of the frequency with which each value was coded within different source material categories, along with the proportion of codes that reference each value in that category.

**Table 2.** Rankings of the number of coding references for each value and the percentage of coding references within each group attributed to that value.

Value	Source material			
	CPG rank (%)	Questionnaires rank (%)	Codes of ethics rank (%)	Total rank, *n*, (%)
Subjective benefit	2 (13.4)	1 (65.5)		**1** 498 (37.2)
Professional duties	6 (7.3)	9.5 (0.3)	1 (75.7)	**2** 225 (16.8)
Self-efficacy	11.5 (3.2)	2 (18.5)		**3** 138 (10.3)
Accuracy	1 (18.3)		7.5 (0.8)	**4** 77 (5.7)
Comfort	3 (9.0)	3 (5.5)		**5** 74 (5.5)
Safety	13 (2.9)	4 (4.0)	6 (1.2)	**6** 42 (3.1)
Objective benefit	4 (8.3)	11.5 (0.1)	7.5 (0.8)	**7** 37 (2.8)
Health	5 (8.1)		9 (0.4)	**8** 34 (2.5)
Autonomy	11.5 (3.2)	8 (0.6)	3.5 (5.5)	**9.5** 31 (2.3)
Satisfaction	9.5 (3.4)	5 (2.5)		**9.5** 31 (2.3)
Ease of use	8 (4.9)	6 (1.8)		**11** 32 (2.4)
Evidence-based	7 (5.9)			**12.5** 24 (1.8)
Equity	15 (2.5)	11.5 (0.1)	3.5 (5.5)	**12.5** 24 (1.8)
Access to care	17 (1.7)		5 (3.5)	**14.5** 16 (1.2)
Privacy	18 (2.2)		2 (5.9)	**14.5** 16 (1.2)
Efficiency	9.5 (3.4)		9 (0.4)	**16** 15 (1.1)
Cost	16 (2.0)	7 (0.7)	9 (0.4)	**17** 14 (1.0)
Design	14 (2.4)	9.5 (0.3)		**18** 12 (0.9)
Total coding references (*N*)	409	676	255	1,340

*Note.* CPG = clinical practice guideline.

**Figure 2.**
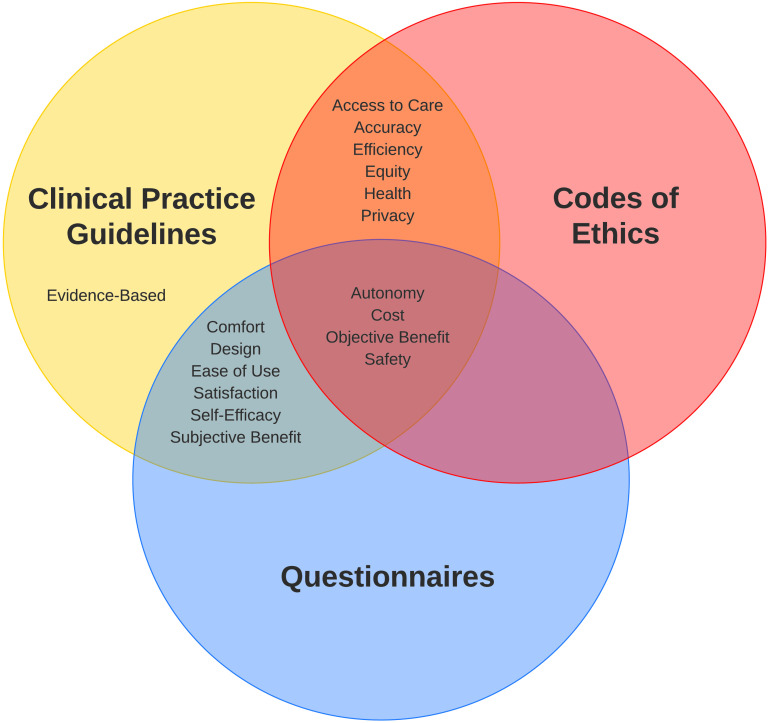
Values coded in each category of source material.

Figure 2 shows where values were found. With the exception of evidence-based, which only appeared in CPGs, all values were identified in at least two out of three of the source material categories. Four values appeared at least once across all categories of source material: autonomy, safety, objective benefit, and cost. All 18 values were referenced in the collected CPGs. When all values are observed in a document category, additional documents in that category are unlikely to reveal new values (Curtis et al., 2001).

## Discussion

The primary result of this study is a comprehensive list of values reflecting the current state of clinical audiology. Compiling this list will facilitate the critical evaluation of service delivery models including OTC and telehealth options and guide development of novel solutions to address disparities in hearing health care access and use. We applied a theoretical framework from VSD to systematically assess the values in source material relevant to hearing health care service delivery as it pertains to the provision of common audiological services in the United States. The source material included questionnaires, CPGs, and codes of ethics documents. These documents are a small subset of all possible documents that could be analyzed, but values not expressed in any of these three types of documents are likely not widespread among audiologists. Directed qualitative content analysis methodology was used to identify 18 concepts representing the intended and enacted values in audiological services.

Subjective benefit, professional duties, and self-efficacy were the most commonly coded values in the documents representing traditional audiology. Together, these three values account for approximately 64% of aggregated words that were coded to a value. The resulting picture of values encoded in professional documents matches with our own understanding of the field as researchers: that the subjective benefits experienced by patients are at the center of our professional guidance (and particularly our evaluation tools) and that these values should be central to considerations in the design of new hearing health care models. At the same time, professional duties currently dominate attention to moral values in the field. If the role of professionals is reduced in future hearing health care delivery models, the field must grapple with how best to preserve attention to moral values. Audiology is a gatekeeping model of health care, where access to treatment (hearing aids) is controlled and limited by those with a professional degree. The emphasis on self-efficacy identified here is consistent with a shift in hearing health care toward empowering adults with perceived difficulty hearing to pursue solutions without the involvement of a health care provider. An OTC model, for example, prioritizes the core value of self-efficacy but would likely de-emphasize the value of professional duties. The identification of key audiology values is the first step toward evaluating the benefits and limitations of alternative service delivery models. By establishing the list of values and their relative importance in traditional audiology, we can evaluate how they are facilitated by current and future products and services.

There are limitations associated with the use of a directed content analysis approach. Using theory to guide the coding process could bias researchers, potentially leading to overly supportive evidence and blinding researchers to contextual aspects of a phenomenon (Hsieh & Shannon, 2005). The influence of researcher bias can affect the neutrality and confirmability of results because the research methods used in the selection and coding of documents are inherently subjective (Lincoln & Guba, 1985). We took steps to achieve unbiased results, including a thorough review of values definitions with all research team members before using the codebook with the source material; a step recommended by Hsieh and Shannon (2005) to increase accuracy of predetermined coding categories. One approach to achieving a neutral interpretation of values would be to have the coding completed by researchers with expertise in values coding and no stake in audiology. Unfortunately, that is not feasible because domain-specific knowledge is required to interpret the source material. Our group consisted of a combination of researchers with expertise in either VSD or audiology, where the audiology expertise was specific to research audiology and clinical training rather than clinical practice. Team members with VSD expertise guided our theoretical framework and the implementation of value-sensitive methodology but were limited in their ability to contribute to coding hearing health care materials due to domain-specific jargon used throughout the reference materials. Given the expertise of our team, we acknowledge our academic positionality and consider that our subjective interpretation of these documents may be biased to reflect the viewpoint of academic researchers. We encourage readers to critically evaluate the list of values published here and to explore how the values relate to their experience of audiology and the extent to which missing values should be included in the field. Another limitation associated with qualitative content analysis has to do with the choice of source material to analyze. We chose to collect CPGs, codes of ethics, and questionnaires that were likely to represent the *intended* and *enacted* values in hearing health care for American adults. It is possible that the documents chosen for this study do not encompass all aspects of hearing health care and that the analysis of additional documents in Step 3 could have produced values that were not captured in our corpus. However, the fact that all codebook values were found in the chosen CPGs is evidence that we would not find new values by including additional CPGs (Curtis et al., 2001).

The purpose of this study was to capture information about core values in audiology as a starting point in the process of using VSD to improve the treatment of hearing loss. The codebook developed in this project was used to capture and interpret information about the values currently prioritized in audiology and can serve as a template to assess the values inherent to hearing health care service delivery. As teleaudiology, OTC, and other hearing health care service delivery models are adopted, this codebook can be used to ensure that important values are preserved or that a shift in values is enacted intentionally to overcome current barriers to treatment. In future work, we hope to guide the development and implementation of innovative solutions that address untreated hearing loss using the codebook presented here.

## Conclusions

A comprehensive list of values representing the field of audiology was obtained using qualitative content analysis. Subjective benefit, professional duties, and self-efficacy were the core values identified in the current provision of audiologic care. VSD has the potential to address barriers to the access and use of hearing health care services that have been identified under the traditional model of audiology. A comprehensive codebook of values is a useful tool for the critical analysis of the shifting regulations, products, and services of hearing health care in the United States.

## Data Availability Statement

The data sets generated and/or analyzed during this study are available from the corresponding author on reasonable request.

## Appendix

### Collected Source Material

The following information is provided to assist readers in locating the documents reviewed in this study. Few documents have an explicit Digital Object Identifier (DOI). Many questionnaires are embedded in manuscripts with a title that does not match the title of the questionnaire. Other documents are difficult to locate without explicit instructions because they do not have a unique title or consistent web address.

Clinical Practice Guidelines

▪  Title: Standards of Practice for Audiology
    Organization: American Academy of Audiology
    Year of publication: 2012
    Web link: https://www.audiology.org/wp-content/uploads/2021/05/StandardsofPractice.pdf_539945677d6235.20360502.pdf
    Instructions: visit http://www.audiology.org ➔ Practice Resources ➔ Practice guidelines and standards ➔ Standards of practice
▪  Title: Guidelines for Hearing Aid Fitting for Adults
    Organization: ASHA Ad Hoc Committee on Hearing Aid Selection and Fitting
    Year of publication: 1998
    Citation: ASHA Ad Hoc Committee on Hearing Aid Selection and Fitting. (1998). Guidelines for hearing aid fitting for adults. *American Journal of Audiology*, *7*(1), 5–13.
    Web link: https://doi.org/10.1044/1059-0889.0701.05
▪  Title: The Independent Hearing Aid Fitting Forum (IHAFF) Protocol
    Authors: Michael Valente and Dennis Van Vliet
    Year of publication: 1997
    Citation: Valente, M., & Van Vliet, D. (1997). The Independent Hearing Aid Fitting Forum (IHAFF) protocol. *Trends in Amplification, 2*(1), 6–35.
    Web link: https://doi.org/10.1177/108471389700200102
▪  Title: Guidelines for the Audiologic Management of Adult Hearing Impairment
    Author: Michael Valente
    Organization: American Academy of Audiology Task Force
    Citation: Valente, M. (2006). Guideline for audiologic management of the adult patient. *Audiology Online*.
    Year of publication: 2006
    Web link: https://www.audiologyonline.com/articles/guideline-for-audiologic-management-adult-966
    Instructions: visit http://www.audiologyonline.com ➔ journal ➔ articles ➔ search “guideline for audiologic management of the adult patient”
▪  Title: Hearing Aid Fitting Standards for Adult and Geriatric Patients
    Organization: Audiology Practice Standards Organization
    Year of publication: 2021
    Web link: https://www.audiologystandards.org/standards/display.php?id=102
    Instructions: visit http://www.audiologystandards.org ➔ current standards ➔ S2.1 Hearing Aid Fitting for Adult & Geriatric Patients

Questionnaires

Title: The Amsterdam Inventory for Auditory Disability and Handicap
    Citation: Meijer, A. G. W., Wit, H. P., & Albers, F. W. J. (2004). Relation between change of hearing and (modified) Amsterdam Inventory for Auditory Disability and Handicap Score. *Clinical Otolaryngology & Allied Sciences*, *29*(6), 565–570.
    Instructions: Questionnaire items found in Appendix 2
    Web link: https://doi.org/10.1111/j.1365-2273.2004.00844.x
Title: Abbreviated Profile of Hearing Aid Benefit
    Citation: Cox, R. M., & Alexander, G. C. (1995). The Abbreviated Profile of Hearing Aid Benefit. *Ear and Hearing*, *16*(2), 176–186.
    Instructions: Questionnaire items found in Appendix A
Title: NAL Client Oriented Scale of Improvement
    Citation: Dillon, H., Birtles, G., & Lovegrove, R. (1999). Measuring the outcomes of a national rehabilitation program: Normative data for the Client Oriented Scale of Improvement (COSI) and the Hearing Aid User's Questionnaire (HAUQ). *Journal of the American Academy of Audiology*, *10*(02), 67–79.
    Web link: https://doi.org/10.1055/s-0042-1748459
Title: The Communication Profile for the Hearing Impaired
    Citation: Demorest, M. E., & Erdman, S. A. (1987). Development of the Communication Profile for the Hearing Impaired. *Journal of Speech and Hearing Disorders*, *52*(2), 129–143.
    Web link: https://doi.org/10.1044/jshd.5202.129
    Instructions: Questionnaire items found in Appendix
Title: Revised Communication Self-Assessment Scale Inventory for Deaf Adults
    Citation: Kaplan, H., Bally, S. J., & Brandt, F. (1995). Revised Communication Self-assessment Scale Inventory for Deaf Adults (CSDA). *Journal of the American Academy of Audiology*, *6*(4), 311–329.
    Instructions: Questionnaire items found in Appendix
Title: Effectiveness of Auditory Rehabilitation Scale
    Citation: Yueh, B., McDowell, J. A., Collins, M., Souza, P. E., Loovis, C. F., & Deyo, R. A. (2005). Development and validation of the effectiveness of auditory rehabilitation scale. *Archives of Otolaryngology—Head & Neck Surgery*, *131*(10), 851–856.
    Instructions: Questionnaire items found in Table 2
    Web link: https://doi.org/10.1001/archotol.131.10.851
Title: Glasgow Hearing Aid Benefit Profile
    Citation: Gatehouse, S. (2000). The Glasgow Hearing Aid Benefit Profile: What it measures and how to use it. *The Hearing Journal*, *53*(3), 10–12.
    Instructions: Questionnaire items found in Appendix B
Title: Gothenburg Profile
    Citation: Ringdahl, A., Eriksson-Mangold, M., & Andersson, G. (1998). Psychometric evaluation of the Gothenburg Profile for measurement of experienced hearing disability and handicap: Applications with new hearing aid candidates and experienced hearing aid users. *British Journal of Audiology*, *32*(6), 375–385.
    Web link: https://doi.org/10.3109/03005364000000089
    Instructions: Questionnaire items found in Appendix
Title: Hearing Aid Needs Assessment
    Citation: Schum, D. J. (1999). Perceived hearing aid benefit in relation to perceived needs. *Journal of the American Academy of Audiology*, *10*(01), 40–45.
    Instructions: Questionnaire items found in Table 2
    Web link: https://doi.org/10.1055/s-0042-1748329
Title: The Hearing Aid Performance Inventory
    Citation: Walden, B. E., Demorest, M. E., & Hepler, E. L. (1984). Self-report approach to assessing benefit derived from amplification. *Journal of Speech and Hearing Research*, *27*(1), 49–56.
    Instructions: Questionnaire items found in Appendix
    Web link: https://doi.org/10.1044/jshr.2701.49
Title: Hearing Attitudes in Rehabilitation Questionnaire
    Citation: Hallam, R. S., & Brooks, D. N. (1996). Development of the Hearing Attitudes in Rehabilitation Questionnaire (HARQ). *British Journal of Audiology*, *30*(3), 199–213.
    Instructions: Questionnaire items found in Table 1
    Web link: https://doi.org/10.3109/03005369609079040
Title: Hearing Aid User's Questionnaire
    Citation: Dillon, H., Birtles, G., & Lovegrove, R. (1999). Measuring the outcomes of a national rehabilitation program: Normative data for the Client Oriented Scale of Improvement (COSI) and the Hearing Aid User's Questionnaire (HAUQ). *Journal of the American Academy of Audiology*, *10*(02), 67–79.
    Web link: https://doi.org/10.1055/s-0042-1748459
Title: The Hearing Coping Assessment
    Citation: Andersson, G., Melin, L., Lindberg, P., & Scott, B. (1995). Development of a short scale for self-assessment of experiences of hearing loss: The Hearing Coping Assessment. *Scandinavian Audiology, 24*(3), 147–154.
    Instructions: Questionnaire items found in Table 1
    Web link: https://doi.org/10.3109/01050399509047528
Title: Hearing Handicap Inventory for Adults
    Citation: Newman, C. W., Weinstein, B. E., Jacobson, G. P., & Hug, G. A. (1990). The Hearing Handicap Inventory for Adults: Psychometric adequacy and audiometric correlates. *Ear and Hearing*, *11*(6), 430–433.
    Instructions: Modifications from the Hearing Handicap Inventory for the Elderly found in Appendix
    Web link: https://doi.org/10.1097/00003446-199012000-00004
Title: Hearing Handicap Inventory for the Elderly
    Citation: Newman, C. W., & Weinstein, B. E. (1988). The Hearing Handicap Inventory for the Elderly as a measure of hearing aid benefit. *Ear and Hearing*, *9*(2), 81–85.
    Instructions: Questionnaire items found in Appendix
    Web link: https://doi.org/10.1097/00003446-198804000-00006
Title: Hearing Handicap Scale
    Citation: High, W. S., Fairbanks, G., & Glorig, A. (1964). Scale for Self-Assessment of Hearing Handicap. *Journal of Speech and Hearing Disorders, 29*(3), 215–230.
    Instructions: Questionnaire items found under “Form A” and “Form B”
    Web link: https://doi.org/10.1044/jshd.2903.215
Title: Hearing Performance Inventory
    Citation: Giolas, T. G., Owens, E., Lamb, S. H., & Schubert, E. D. (1979). Hearing Performance Inventory. *Journal of Speech and Hearing Disorders*, *44*(2), 169–195.
    Instructions: Questionnaire items found in Appendix B
    Web link: https://doi.org/10.1044/jshd.4402.169
Title: The International Outcome Inventory for Hearing Aids
    Citation: Cox, R. M., & Alexander, G. C. (2002). The International Outcome Inventory for Hearing Aids (IOI-HA): Psychometric properties of the English version. *International Journal of Audiology*, *41*(1), 30–35.
    Web link: https://doi.org/10.3109/14992020209101309
    Instructions: Questionnaire items found in Appendix
Title: MarkeTrak V Customer Satisfaction for Hearing Instruments
    Citation: Kochkin, S. (2005). MarkeTrak VII: Customer satisfaction with hearing instruments in the digital age. *The Hearing Journal*, *58*(9), 30–32.
    Instructions: Questionnaire items found in Table 1
    Web link: https://doi.org/10.1097/01.HJ.0000286545.33961.e7
Title: Profile of Hearing Aid Performance
    Citation: Cox, R. M., & Gilmore, C. (1990). Development of the Profile of Hearing Aid Performance (PHAP). *Journal of Speech and Hearing Research*, *33*(2), 343–357.
    Web link: https://doi.org/10.1044/jshr.3302.343
    Instructions: Questionnaire items found in Appendix
Title: Performance Inventory for Profound and Severe Loss
    Citation: Owens, E., & Raggio, M. (1988). Performance Inventory for Profound and Severe Loss (PIPSL). *Journal of Speech and Hearing Disorders*, *53*(1), 42–56.
    Instructions: Questionnaire items found in Appendix C
    Web link: https://doi.org/10.1044/jshd.5301.42
Title: Patient Self-Assessment of Communication
    Citation: Ivory, P. J., Hendricks, B. L., Van Vliet, D., Beyer, C. M., & Abrams, H. B. (2009). Short-term hearing aid benefit in a large group. *Trends in Amplification*, *13*(4), 260–280.
    Instructions: Questionnaire items found in Appendix C
    Web link: https://doi.org/10.1177/1084713809354902
Title: Satisfaction with Amplification in Daily Life
    Citation: Cox, R. M., & Alexander, G. C. (1999). Measuring Satisfaction with Amplification in Daily Life: The SADL scale. *Ear and Hearing, 20*(4), 306–320.
    Web link: https://doi.org/10.1097/00003446-199908000-00004
    Instructions: Questionnaire items found in Appendix B
Title: The Speech, Spatial, and Qualities of Hearing Scale
    Citation: Gatehouse, S., & Noble, W. (2004). The Speech, Spatial and Qualities of Hearing Scale (SSQ). *International Journal of Audiology*, *43*(2), 85–99.
    Web link: https://doi.org/10.1080/14992020400050014
    Instructions: Questionnaire items found in Appendix 2

Codes of Ethics

Title: Code of Ethics of the American Academy of Audiology
    Organization: American Academy of Audiology
    Year of publication: 2021
    Web link: https://www.audiology.org/wp-content/uploads/2021/05/201910-CodeOfEthicsOf-AAA.pdf
    Instructions: visit http://www.audiology.org ➔ Practice Resources ➔ Practice guidelines and standards ➔ Code of Ethics
Title: Academy of Doctors of Audiology Code of Ethics
    Organization: Academy of Doctors of Audiology
    Web link: https://www.audiologist.org/_resources/documents/about/academy-docs/Code-of-Ethics.pdf
    Instructions: visit http://www.audiologist.org ➔ About Us ➔ Academy Documents ➔ Code of Ethics
Title: American Speech-Language-Hearing Association Code of Ethics
    Organization: ASHA
    Year of publication: 2016
    Citation: American Speech-Language-Hearing Association. (2016). *Code of ethics* [Ethics].
    Web link: https://inte.asha.org/Code-of-Ethics/
    Instructions: visit http://www.asha.org ➔ Practice Policy ➔ Ethics ➔ Code of Ethics
Title: Code of Ethics of the National Board for Certification in Hearing Instrument Sciences
    Organization: NBC-HIS
    Year of publication: 2013
    Web link: https://www.nbc-his.com/code-of-ethics/
    Instructions: visit http://www.nbc-his.com ➔ Hearing health care professionals ➔ About ➔ Code of Ethics
